# The Compact Macronuclear Genome of the Ciliate Halteria grandinella: A Transcriptome-Like Genome with 23,000 Nanochromosomes

**DOI:** 10.1128/mBio.01964-20

**Published:** 2021-01-26

**Authors:** Weibo Zheng, Chundi Wang, Michael Lynch, Shan Gao

**Affiliations:** a Institute of Evolution & Marine Biodiversity, Ocean University of China, Qingdao, China; b Biodesign Institute, Center for Mechanisms of Evolution, Arizona State University, Tempe, Arizona, USA; c School of Life Sciences, Ludong University, Yanti, China; d Laboratory for Marine Biology and Biotechnology, Qingdao National Laboratory for Marine Science and Technology, Qingdao, China; Smith College; University of Southern California

**Keywords:** alternative DNA splicing, ciliate, *Halteria grandinella*, nanochromosomes, transcriptional initiation

## Abstract

How to achieve protein diversity by genome and transcriptome processing is essential for organismal complexity and adaptation. The present work identifies that the macronuclear genome of Halteria grandinella, a cosmopolitan unicellular eukaryote, is composed almost entirely of gene-sized nanochromosomes with extremely short nongenic regions. This challenges our usual understanding of chromosomal structure and suggests the possibility of novel mechvanisms in transcriptional regulation. Comprehensive analysis of multiple data sets reveals that *Halteria* transcription dynamics are influenced by: (i) nonuniform nanochromosome copy numbers correlated with gene-expression level; (ii) dynamic alterations at both the DNA and RNA levels, including alternative internal eliminated sequence (IES) deletions during macronucleus formation and large-scale alternative splicing in transcript maturation; and (iii) extremely short 5′ and 3′ untranslated regions (UTRs) and universal TATA box-like motifs in the compact 5′ subtelomeric regions of most chromosomes. This study broadens the view of ciliate biology and the evolution of unicellular eukaryotes, and identifies *Halteria* as one of the most compact known eukaryotic genomes, indicating that complex cell structure does not require complex gene architecture.

## Abstract (precis)

How to achieve protein diversity by genome and transcriptome processing is essential for organismal complexity and adaptation. The present work identifies that the macronuclear genome of Halteria grandinella, a cosmopolitan unicellular eukaryote, is composed almost entirely of gene-sized nanochromosomes with extremely short nongenic regions.

## INTRODUCTION

Ciliates are regarded as one of the most diverse and highly differentiated groups of unicellular eukaryotes, based on their nuclear dimorphism (each cell contains two types of nuclei, a germ line micronucleus and a somatic macronucleus), complex epidermal ciliation, and strong swimming ability (1, 2). These features make ciliates excellent models for research in cell biology and evolution (3–8), leading to many important biological discoveries, including catalytic RNA, telomerase, and histone acetylation (9–11).

*Halteria grandinella* is a highly specialized ciliate distributed worldwide and very common in all freshwater ecosystems (12–14). It can be easily distinguished from other ciliates by its special “jumping” motion, remaining stationary for a while and then swimming rapidly backward at a speed of 100 body lengths per second (13, 15). Since the first report of *Halteria grandinella* by Mueller (1773), much research on morphology, taxonomy, ecology, and cell biology has been performed (12–16), illustrating the potential of this organism as a model for biological studies.

**FIG 1:**
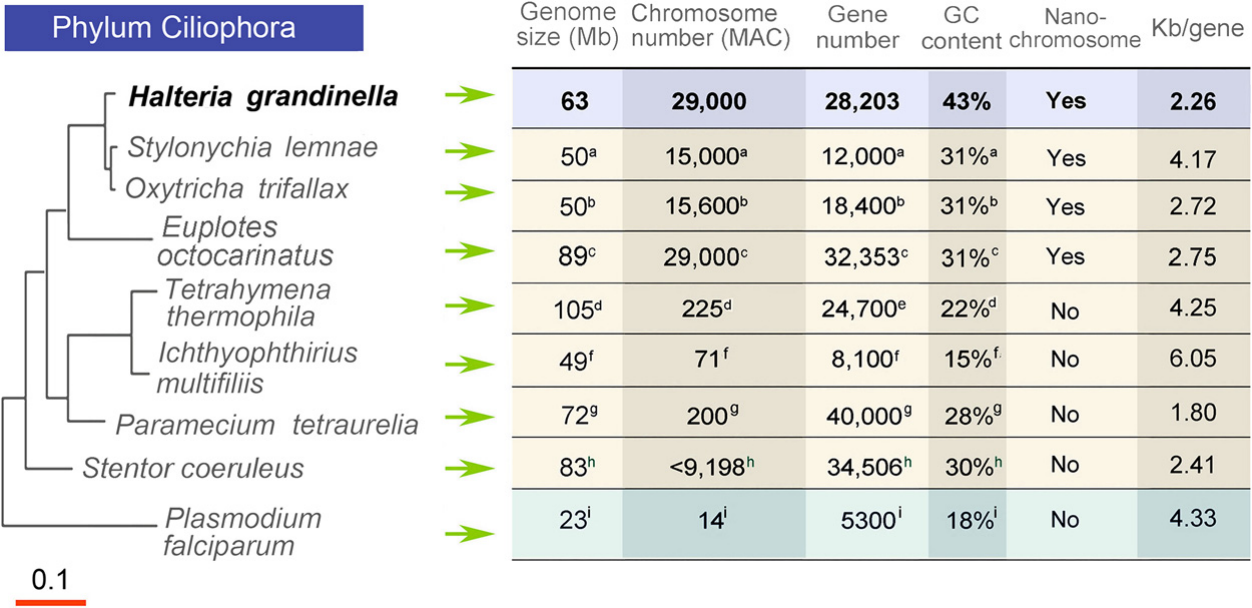
Comparison of macronuclear genome features. The molecular phylogenetic relationship was constructed by using the maximum likelihood method based on MUSCLE multiple-sequence alignment of 18S rRNA genes (*Halteria*, MF002432.1; *Stylonychia lemnae*, AM233915.1; *Oxytricha trifallax*, FJ545743.1; *Euplotes octocarinatus*, LT623905.1; Tetrahymena thermophila, M10932.1; Ichthyophthirius multifiliis, U17354.1; Paramecium tetraurelia, AB252009.1; *Stentor coeruleus*, JQ282899.1; and an outgroup Plasmodium falciparum, MF155937.1). Genome statistics for *Halteria* were determined in this study. Other statistics were obtained from the following sources: a, reference 25; b, reference 20; c, reference 77; d, reference 18; e, reference 78; f, reference 79; g, reference 19; h, reference 56; i, reference 80. The scale bar corresponds to 0.1 expected substitutions per nucleotide site.

Unlike the well-studied ciliate models whose macronuclear genomes are slightly fragmented into ∼200 and ∼800 chromosomes (17–19), such as *Tetrahymena* and *Paramecium*, *Halteria* possesses a polyploid macronuclear genome (63 Mb haploid genome size with ∼23,000 chromosomes) with a 2,800-bp mean chromosome size (Fig. 1). This extremely fragmented macronuclear genome resembles the macronuclear genome of Oxytricha trifallax (16,000 nanochromosomes with a mean length of ∼3,200 bp) (20). Nanochromosomes in *Halteria* are capped with telomeres on both ends (5′ telomere [C4A4]_2–3_C4) and usually only code for a single gene. Even the smallest chromosome in *Oxytricha* (430 bp) possesses a single well-positioned nucleosome (21), suggesting that transacting factors, such as ATP-dependent chromatin remodelers, are involved in transcription regulation in nanochromosomes. Due to this one-gene one-chromosome feature (22), the colinear structure of multiple genes common to almost all eukaryotic organisms is greatly limited, further restricting the definition of a conventional “intergenic region.” The intergenic regions in most eukaryotes are non-coding regions surrounded by two genes and usually harbor numerous regulatory components, such as promoters and enhancers (23, 24). In contrast, most noncoding regions (except introns) of *Halteria* are very short and directly flanked by telomeres rather than genes. These distinct features of the *Halteria* macronuclear genome suggest the possibility of novel mechanisms in the regulation of transcriptional processes.

To comprehensively understand the transcriptional dynamics in this highly compact genome, we carried out deep genomic sequencing to reveal the first macronuclear genome sequences of *Halteria* with gene prediction and functional annotation. Furthermore, by integrating genome and transcriptome data sets, we addressed and analyzed several challenges arising from this highly fragmented genome. This research broadens the view of ciliate biology and the evolution of unicellular eukaryotes, and identifies *Halteria* as one of the most compact known eukaryotic genomes, indicating that complex cell structure does not require complex gene architecture.

## RESULTS AND DISCUSSION

### Transcriptome-like genome.

**FIG 2:**
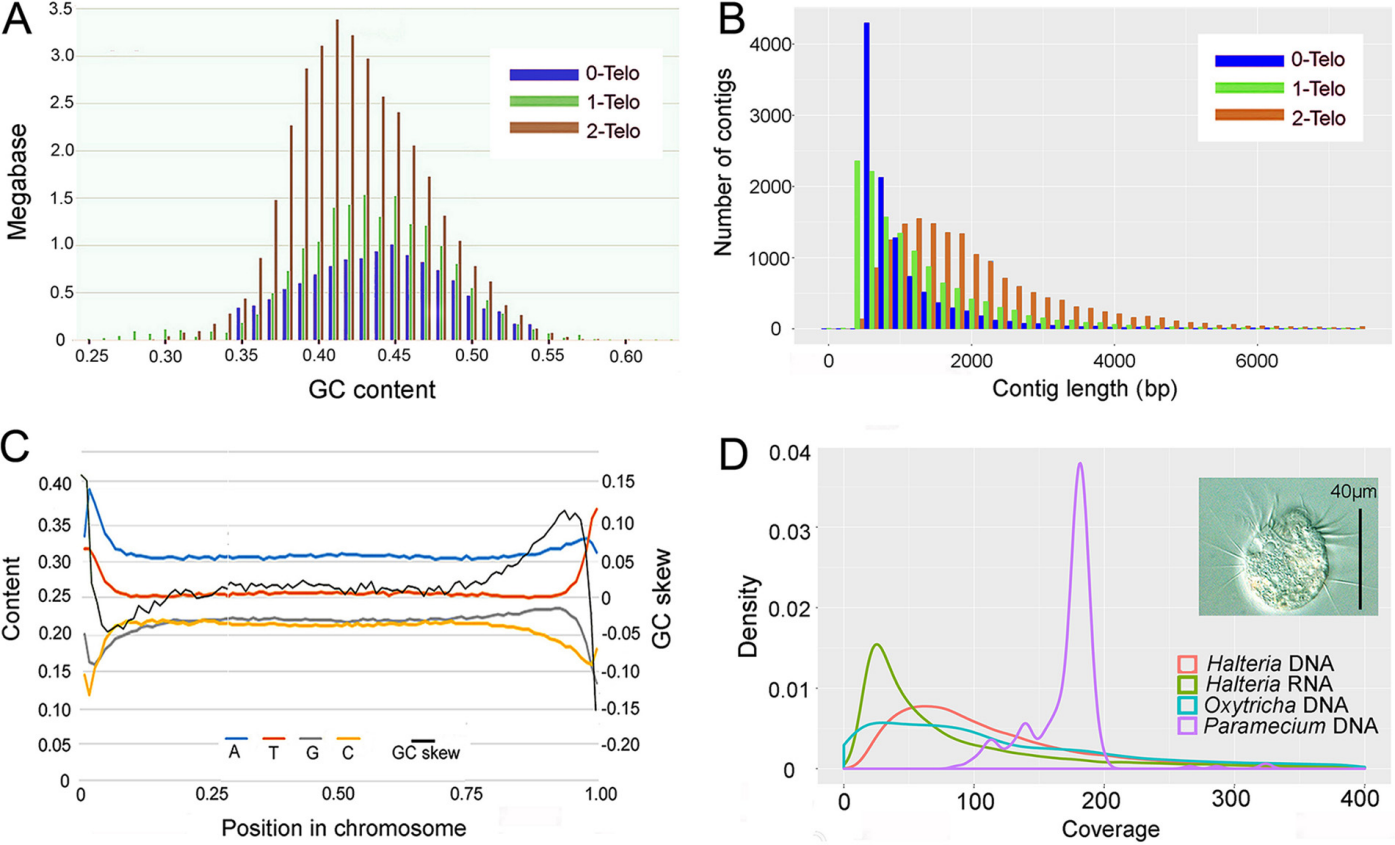
Features of the transcriptome-like genome of *Halteria*. (A) Distribution of GC content across total lengths of 0-, 1-, and 2-telomere contigs. (B) Length distribution of contigs with 0, 1, and 2 telomeres. (C) Sliding-window analysis of nucleotide content. Window size was 1% of the whole chromosome, excluding telomeres. Only the coding strands of single-gene coding chromosomes were used. GC skew: (G−C)/(G+C). (D) Distribution of RNA coverage level of all contigs in *Halteria*, and distribution of DNA coverage level for all contigs in *Halteria*, *Oxytricha*, and *Paramecium* genomes (density plot, with the area under each curve normalized to 1.0). All data sets were normalized to the same average coverage level by controlling the total reads number. A microphotograph of a living *Halteria* is shown.

**The macronuclear (MAC) genome of *Halteria* is composed of nanochromosomes.** After removal of low-quality and nearly identical contigs, mitochondrial contigs (four, summing to 50 kb), and contamination from bacterial genomes, the final assembly of the *Halteria* macronuclear genome is ∼64 Mb and includes 40,570 contigs. Only 13 out of 40,570 contigs have non-self matches that are ≥99% identical (matches that are ≥100 bp), indicating that the assembled genome is haploid. The haploid size of the *Halteria* MAC genome is similar to that of other ciliates with nanochromosomes, such as *Oxytricha*, *Stylonychia*, *Euplotes*, *Nyctotherus*, *Urostyla*, *Paraurostyla*, and *Tetmemena*, each of which has a 50 to 70 Mb MAC genome (20, 25–28) (Fig. 1). A total of 16,506 contigs (∼35 Mb) of this assembly have telomeres on both ends, each representing a complete MAC chromosome (Table 1). Contigs with one telomere (13,251, totaling ∼17 Mb) and zero telomeres (10,813, totaling ∼12 Mb) comprise less than half of the whole assembly (Fig. 2A). The estimated haploid chromosome number of the MAC genome is 23,131 (half the number of one-telomere contigs plus the number of two-telomere contigs). The overall GC content of all contigs, 43.11% (42.91% for all two-telomere chromosomes), is relatively high compared with other ciliates (18, 20, 29). The final assembly was verified by BLAST against bacterial genomes to ensure there was no bacterial contamination from the food source of *Halteria* (30) and was estimated to contain negligible MIC sequence contamination by comparing the reads coverage of adjacent regions (31). Structural variation of the high ploidy MAC chromosomes, however, will be resolved in the future by long-read sequencing technology such as single molecule, real-time (SMRT) sequencing (3).

**TABLE 1:** Statistical details of genome assembly

Parameter^*a*^	Two-telomere contigs	One-telomere contigs	Zero-telomere contigs	Total
No. all contigs	16,506	13,251	10,813	40,570
No. contigs ≥ 1 kb	13,582	6,412	3,661	23,655
No. contigs ≥ 5 kb	689	215	100	1,004
No. contigs ≥ 10 kb	57	20	23	100
No. contigs ≥ 25 kb	1	1	0	2
No. contigs ≥ 50 kb	1	0	0	1
Largest contig (bp)	74,041	41,562	17,665	74,041
Total length (bp)	34,693,884	17,510,270	11,948,065	64,152,219
*N*_50_ (bp)	2,485	1,767	1,214	2,064
*N*_75_ (bp)	1,653	1,029	752	1,232
*L*_50_ (no.)	4,407	2,952	2,668	9,385
*L*_75_ (no.)	8,706	6,221	5,855	19,399
GC (%)	42.59	43.53	44.00	43.11

a *N*_50_/*N*_75_, the length of the contig at 50/75% of the cumulative total length of the whole genome. *L*_50_/*L*_75_, the smallest number of contigs whose length sum produces *N*_50_/*N*_75_.

If we focus on the well-assembled chromosomes (2-telomere contigs), we see an assembly of tiny chromosomes. Including the length of telomeres (20 to 28 bp on each chromosome end), the *N*_50_ length (the length of the contig at 50% of the cumulative total length of the whole genome) of two-telomere capped chromosomes is just 2,485 bp, which is close to the length of a regular transcript (below). This length is much smaller than that found in *Oxytricha trifallax* (3,392 bp, strain JRB510), which has a similar well-assembled highly fragmented macronuclear genome (28). The distribution of chromosome lengths indicates that ∼74% of all two-telomere chromosomes have lengths of <2,500 bp, with 138 having lengths of <500 bp. The longest was 74,042 bp and the shortest was 345 bp (Fig. 2B).

**Gene prediction and genome annotation.** A total of 28,203 genes were predicted, 16,267 of which have functionally annotated orthologs in other species, with the remaining 11,936 predicted as hypothetical protein-coding genes without functional information. A total of 11,800 chromosomes contain at least one gene, most of them (77%) only encoding a single gene (Fig. S1 in the supplemental material). The gene number in each contig correlates with the length of the contig (mean lengths of contigs containing 1, 2, and 3 genes: 1,643, 2,748, and 3,595 bp). However, the longest complete chromosome, which is 74,042 bp in length, contains only a single gene (length: 63,864 bp) with best hits to *titin*-like genes in the Swiss-Prot database (32). Part of this gene has high similarity (total length of the matched region is 26,187 bp, with 58% identity) with the longest gene of *Oxytricha trifallax* (located in contig7580 of this species and denoted “Jotin”), which is 64,614 bp in length and also encoded in the longest chromosome (65,957 bp) (20).

10.1128/mBio.01964-20.1 FIG S1 The distribution of numbers of genes in gene-coding chromosomes (2-telomere contigs). Download FIG S1, TIF file, 1.2 MB. Copyright © 2021 Zheng et al. 2021 Zheng et al. This content is distributed under the terms of the Creative Commons Attribution 4.0 International license.

Sliding-window analyses of the coding strands of single-gene chromosomes reveal spatial variation in nucleotide content (Fig. 2C). On average, the transcribed region is slightly adenine rich (A: ∼31%; T: ∼26%), and there is an obvious GC skew, measured as (G−C)/(G+C), in the 3′ subtelomeric region of the coding strand. A similar phenomenon was previously noted in *Oxytricha*, but we could not detect the ∼10-bp periodicity reported therein (33). A potential explanation for the strand-sensitive GC bias in 3′ subtelomeric regions of coding strands is that, unlike the transcribed anti-sense strand, which is used as a template and protected by the transcription complex, the nontranscribed coding strand is single-stranded during transcription. When exposed as a single strand, cytosine is prone to deamination to uracil, resulting in errors in newly synthesized strands (33, 34). Under this hypothesis, compared with the 5′ subtelomeric region, which may be renatured in a shorter time after the initiation of transcription (35), and the coding region, which is under stronger selective pressure, the 3′ subtelomeric region may be more vulnerable to the accumulation of deamination-associated mutations (34), causing cytosines to be replaced by guanines, forming the GC skew. We also analyzed the base composition in the subtelomeric region (Fig. S2), however, we did not detect the ∼10-bp periodicity reported in *Oxytricha* (33). Nonetheless, there is a high similarity between the base compositions of forward and reverse strands, which is consistent with the speculation that this skewed base composition is not a transcription-related outcome (33).

10.1128/mBio.01964-20.2 FIG S2 Shannon entropy (H) composition of each nucleotide for the first 200 positions of all single-gene coding chromosomes. (A) All chromosomes. (B) Chromosomes with blast hits in the forward direction. (C) Chromosomes with blast hits in the reverse direction. Download FIG S2, TIF file, 1.3 MB. Copyright © 2021 Zheng et al. 2021 Zheng et al. This content is distributed under the terms of the Creative Commons Attribution 4.0 International license.

**Copy numbers of macronuclear chromosomes are correlated with expression level.** By mapping the Illumina sequencing reads back to the genome assembly, we obtained an average sequencing coverage of contigs of 108×. At such high coverage, if the copy numbers of macronuclear chromosomes of *Halteria* are uniform, the coverage distribution should be approximately Gaussian in form, with deviations largely resulting from random errors in sequencing and assembly. However, the distribution of sequencing coverage of contigs, which is expected to reflect chromosomal copy numbers, is strongly skewed, much like the typical distribution of transcriptome sequencing (RNA-seq) reads coverage (reflecting wide-range expression levels) in most species (36) (Fig. 2D). This suggests that in the polyploid macronuclear genome of *Halteria*, copy numbers of each nanochromosome are nonuniform, with a range of 400× between the low- and high-copy-number chromosomes (Fig. 2D). We also performed the same analysis on *Oxytricha trifallax* (genome assembly: GCA_000295675.1, genome reads: SRX955799) and Paramecium tetraurelia (genome assembly: GCA_000165425.1, genome reads: ERX2814584) (Fig. 2D). *O. trifallax* has highly fragmented nanochromosomes and nonuniform chromosome copy numbers as in *Halteria* (20), whereas P. tetraurelia has 200 chromosomes with more uniform copy numbers (800 to 1,000 copies for each chromosome) (37). As shown in Fig. 2D, *Paramecium* shows a significantly different copy number distribution than the skewed distributions in *Halteria* and *Oxytricha*.

This kind of nonuniform copy number is potentially relevant to the transcriptome. By mapping the RNA-seq reads to the reference genome, a positive correlation between genome copy number and expression level is revealed. The Spearman’s rank-order correlation coefficient between the genome copy number and average expression level of four RNA-seq replicates is ∼0.56 (*P* value < 2.2e−16). Thus, chromosomes with higher copy numbers tend to have higher expression levels. For *O. trifallax*, which also has nanochromosomes and positively correlated copy number and expression level (20), Khurana et al. (38) found that 21-nt (nucleotide) small noncoding RNAs (ncRNAs) play important roles in controlling the copy numbers of chromosomes. These small ncRNAs have target biases toward transcription start sites and may directly or indirectly activate mRNA expression. Although the detailed regulatory functions of small ncRNAs in ciliates with highly fragmented macronuclear genomes is still unknown, based on these observations, we speculate that *Halteria* may utilize similar mechanisms as *O. trifallax*, whereby small ncRNAs play a role in regulating both chromosomal copy number and expression level, resulting in the positive correlation between them. However, because the partitioning of macronuclear chromosomes occurs by an unknown amitotic mechanism, rather than by mitosis, we cannot rule out the possibility that much of the covariance in chromosome copy number and gene expression level is a simple consequence of cumulative drift of chromosomal copies during asexual propagation (39–42), rather than a direct product of regulation.

Sequence conservation and copy number are reported to be correlated in some eukaryotes, such as *Paramecium* (19, 43). To investigate whether this is the case in *Halteria*, we compared the number of genes that are successfully mapped with the Clusters of Orthologous Groups (COG) database in 100 quantiles ranked from low to high by their copy numbers. Genes with the top 10% highest copy numbers have obviously higher mapping rate within the COG database (Fig. S3). However, the mapping rate remained at essentially the same low level in the first 90 quantiles, covering a wide range of copy numbers (Fig. S3). Our results thus suggested that gene copy number is less likely to be associated with sequence conservation in *Halteria*.

10.1128/mBio.01964-20.3 FIG S3 Copy number and number of genes successfully matched to COG database. Each bin contains 280 genes. Download FIG S3, TIF file, 0.3 MB. Copyright © 2021 Zheng et al. 2021 Zheng et al. This content is distributed under the terms of the Creative Commons Attribution 4.0 International license.

**Chromosomal drift during asexual reproduction in clonal culture.** Contrary to the situation in species with normal mitotic reproduction, the high numbers chromosome copies in ciliate macronuclei are distributed to progeny in a nonmitotic fashion, leading to the long-term divergence of allelic copies from a 1:1 ratio during clonal propagation (41), blurring the conventional definition of heterozygosity, which we here define as simply implying the presence of two variants at a nucleotide site. Macronuclear mutation during asexual growth may also contribute transiently to the heterozygosity of ciliate MAC genomes (43, 44). By conservatively taking 0.05 to be a cutoff for the minimum frequency of a heterozygous site (with a minimum total coverage of each site of 30×, and for the minor allele of 5×) to guard against false-positives from sequencing errors, we attempted to identify the full spectrum of heterozygous sites. This leads to the identification of 0.96% (616,991) of sites as heterozygous (0-fold redundant sites = 0.48%; 2-fold sites = 1.50%; 4-fold sites = 2.54%; introns = 1.52%; untranslated subtelomeric regions without telomeres = 1.02%).

**FIG 3:**
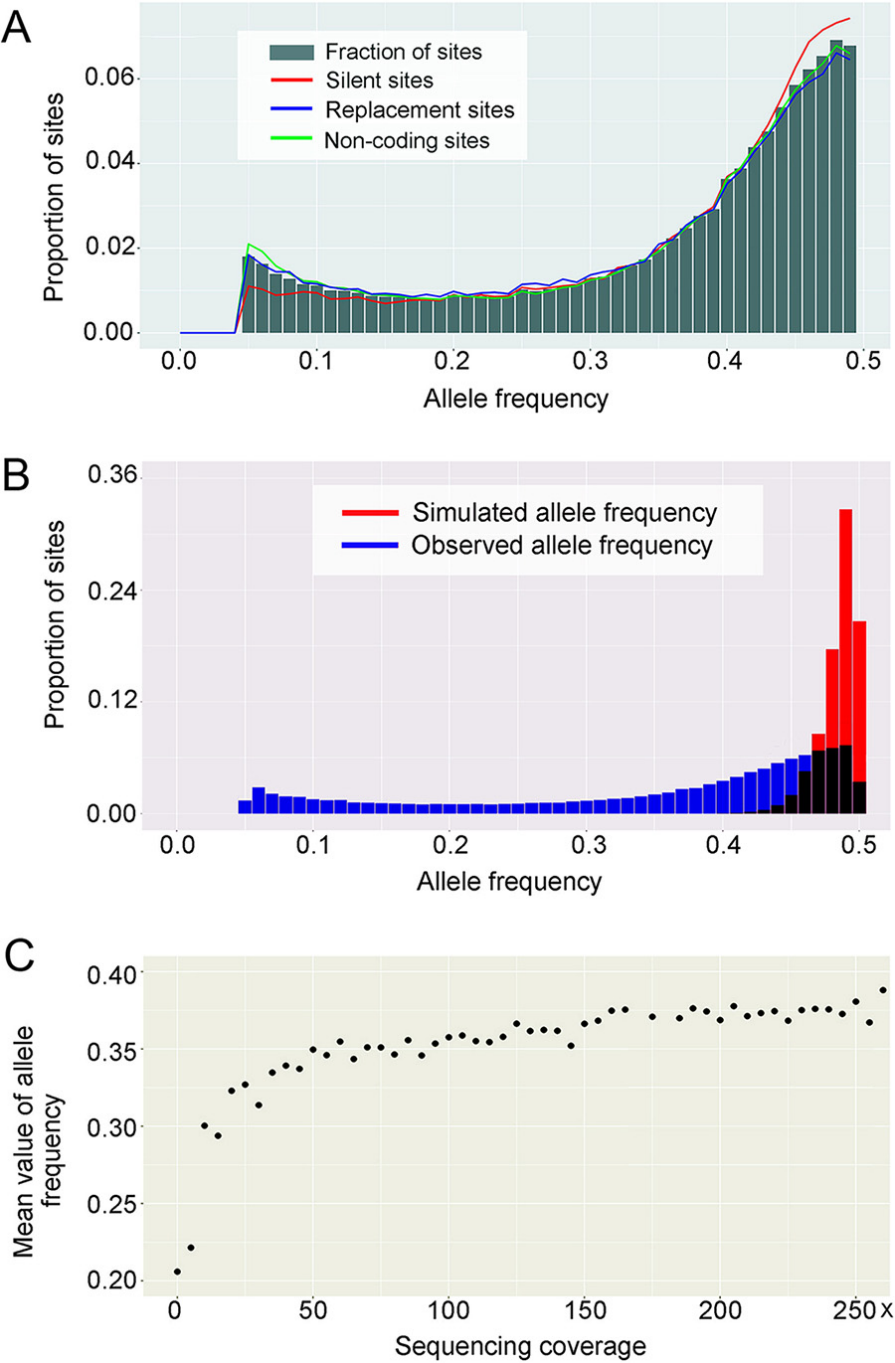
Polymorphic sites in nanochromosomes. (A) Distribution of minor-allele frequency (MAF) for polymorphic sites. The bin size is 0.01. (B) Distributions of allele-frequency estimates and observations. The bin size is 0.01. (C) The mean value of the MAF (gradually increasing) as a function of total sequencing coverage. All heterozygous sites are divided into 50 groups based on the sequencing coverage of chromosomes. Each dot represents a window size of 5× coverage.

Theoretically, if all alleles divided equally into the genomes of daughter cells after each asexual division, alleles should have average frequencies of 0.5 (with variation around this expectation resulting from random sampling of parental chromosomes). This is consistent with our observation that the distribution of minor-allele frequencies peaks at 0.5 (Fig. 3A), but this distribution is also widely distributed from 0.05 to 0.5, with a minor peak at 0.05. The pattern remains after increasing the coverage cutoff for each heterozygous site to 100×, indicating that sequencing error is not the cause of the wide distribution of allele frequencies. We simulated the expectation of this distribution under the assumption of conventional binomial sampling of heterozygous sites assumed to all have 0.5 frequencies (supplemental material appendix, Code S1), demonstrating that the wide distribution seen in *Halteria* is incompatible with simple random sequencing variation (Fig. 3B).

This wide variation of allele frequency of heterozygous sites is likely a result of chromosomal copy drift. Under this view, during asexual reproduction, the macronuclear chromosomes of parental *Halteria* cells are partitioned unequally into daughter cells, gradually increasing deviations from the initial 1:1 ratio of allele pairs expected following the last sexual reproduction event. Such asexual chromosomal copy drift is expected to increase with the number of clonal cell divisions, explaining why clonal lab strains of many ciliates cannot be maintained for long periods (chromosomal copy drift may reduce cell fitness) (45).

As demonstrated in the previous section, the macronuclear genome of *Halteria* is composed of polyploid nanochromosomes with nonuniform copy numbers. In support of the chromosomal-drift hypothesis, there is a positive correlation between the estimated allele frequencies and sequencing coverage (reflecting the copy numbers of chromosomes; Fig. 3C), with heterozygous sites on high-copy chromosomes tending to have estimated frequencies closer to 0.5. As larger deviations in allele frequencies are expected with smaller chromosomal pools, we conclude that chromosomes that have drifted to the smallest copy numbers also experience the highest levels of allele-frequency drift. Our observations are consistent with previous theory on the stochastic drift process and senescence in ciliates by Kimura (40), Preer (39), and Duerr et al. (41). Similar observations were also reported in genome research of *Oxytricha trifallax* (20), indicating that the chromosomal drift is common in ciliates with highly fragmented macronuclear genomes.

### Genome and transcriptome processing increases the diversity of proteins.

**Cryptically excised IESs in nanochromosomes.** During the formation of the highly fragmented macronuclear genome, DNA segments of the micronuclear genome called IESs (internally eliminated sequences) are eliminated (46). These DNA segments are distinguished by their “pointer-sequence” structures, consisting of small nucleotide motifs repeatedly present at the beginning and end of each IES (47, 48). Previous researchers have reported that IESs of ciliates can be alternatively excised, with some macronuclear chromosomes having different versions in the same cell (49–51). Although we do not have a micronuclear genome sequence for *Halteria*, we have designed the ADFinder (Accurate Deletion Finder) software that can detect alternative DNA deletion events in ciliates (52) without the support of micronuclear reads.

By mapping all genomic sequencing reads to the macronuclear genome assembly, we inferred 2,172 alternatively excised IESs with pointer-sequence structure using ADFinder (total length is 299,539 bp). These alternatively excised IESs reflect rare and alternative DNA splicing events during the macronuclear genome development. The average length of these IESs is 101 bp (Fig. S4). Chromosomes containing these alternatively excised IES have multiple isoforms (excised version and nonexcised version). Contrary to our expectations, we found that the average copy number of the excised version was 5.7% of that of the nonexcised version, indicating that the latter is the major version of alternatively excised IES. This is similar to the case discovered in Paramecium tetraurelia (49), in which a majority of cryptically excised IESs are not excised. The majority of alternatively excised IES being nonexcised in *Halteria* reflects the variability of the MAC DNA rather than the contamination of the MIC (micronuclear) DNA, as the latter would have rendered the nonexcised version (the default status of MIC) as the minor form.

10.1128/mBio.01964-20.4 FIG S4 Length distribution of 2,172 alternatively excised IESs in the *Halteria* genome. Download FIG S4, TIF file, 0.5 MB. Copyright © 2021 Zheng et al. 2021 Zheng et al. This content is distributed under the terms of the Creative Commons Attribution 4.0 International license.

Among 2,172 cryptically excised IESs, only 370 (17%) are contained in coding regions, suggesting that cryptically excised IESs are enriched in flanking regions (noncoding regions) of nanochromosomes. The major nonexcised versions in coding regions are highly expressed, with the average RNA-seq coverage at 324×. More strikingly, their minor excised versions are also successfully transcribed, with 75% of them having successfully mapped RNA-seq reads (average RNA-seq coverage = 30×, minimal RNA coverage ≥ 3.6×).

Among those IESs retained in coding regions, 227 of 370 (61.3%) are have lengths that are multiples of 3 (3n IESs) (67.8% for IESs inserted between codons) (Table 2), much more than the random expectation of 33.3%, suggesting that cryptically excised IESs in protein-coding regions tend to retain the original reading frame. In support of this idea, 171 of 370 (46%) IESs in coding regions are inserted between codons, and only a small portion (6.4%) would cause premature stops. For those IESs inserted after the first (97 of 370) and second (102 of 370) nucleotides of codons, 24.3% contain in-frame stop codons. This suggests that some cryptically excised IESs may cause premature translation termination, but most IESs retained in coding regions will not disrupt the original open reading frames (ORFs). In contrast, in *Paramecium* species, cryptically excised IESs are not enriched in coding regions (19% for Paramecium sexaurelia, 28% for Paramecium biaurelia, and 25% for P. tetraurelia) (53). For those cryptically excised IESs in coding regions in *Paramecium*, their length is not inclined to 3n (26% for *P. sexaurelia*, 34% for *P. biaurelia*, and 27% for P. tetraurelia) (53); therefore, the retention of these non-3n IESs will disrupt ORFs and cause premature stops (49, 53, 54).

**TABLE 2:** Cryptically excised IESs in protein-coding regions

Parameter	IESs between codons^*a*^	IESs at frame +1^*a*^	IESs at frame +2^*a*^	Total no.
IESs in protein-coding regions	171	97	102	370
IESs without frameshifts	116	58	53	227
IESs with in-frame stop codons	11	49	30	90

a IESs between codons, cryptically excised IESs inserted between codons; IESs at frame +1, cryptically excised IESs inserted after the first nucleotide of codons; IESs at frame +2, cryptically excised IESs inserted after the second nucleotide of codons.

**FIG 4:**
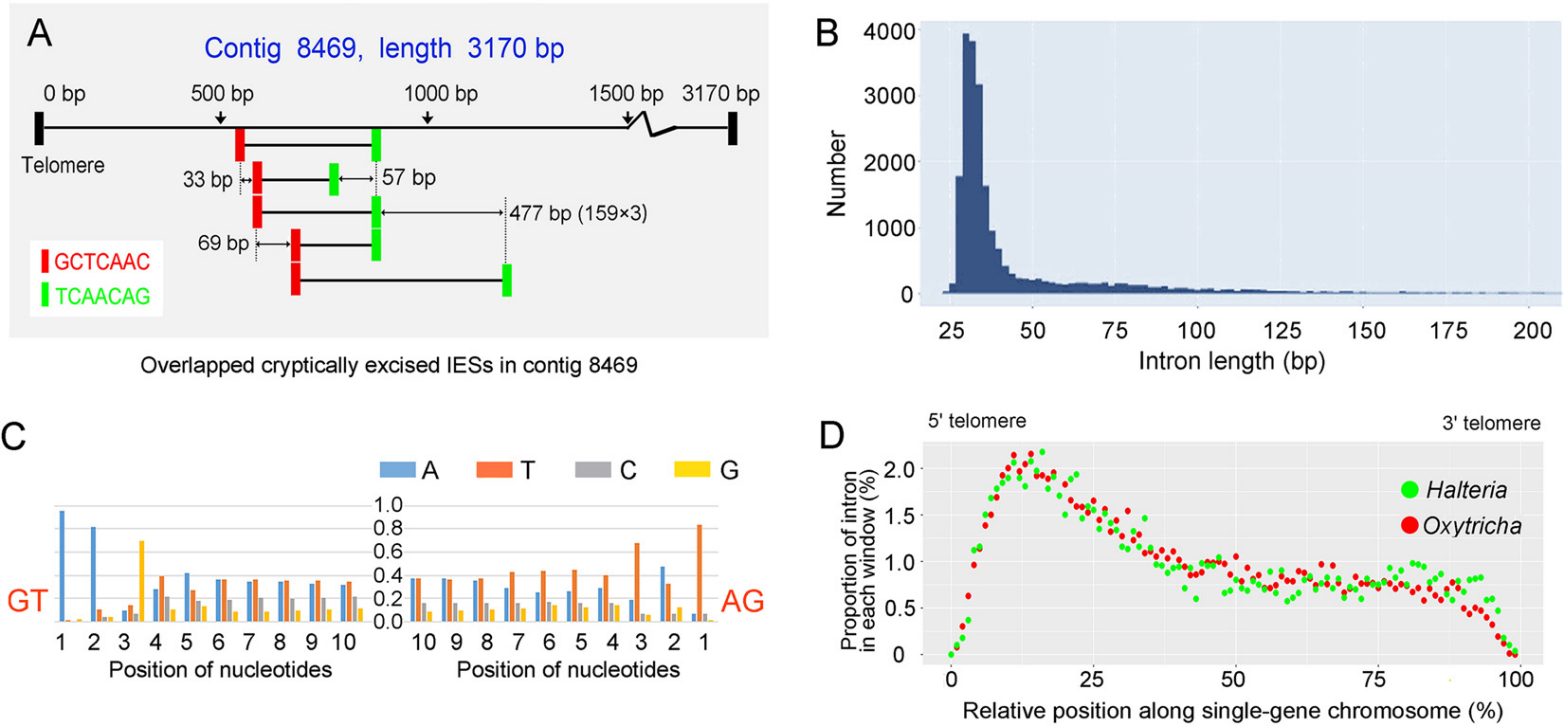
Large-scale DNA and RNA alternative splicing. (A) Cryptically excised IESs in contig 8469. Repetitive segments (each one is 7 bp) are shown in the same color. The length differences between each pair of jointly used segments are shown beside each segment. (B) Length distribution of introns, using bin size of 2 bp. (C) Patterns of nucleotide usage for the 10-bp segment internal to both ends of introns. (D) Distribution of introns in all single-gene coding chromosomes of *Halteria grandinella* and *Oxytricha trifallax*. Window size is 1% of the whole chromosome without two telomeres.

Intriguingly, cryptically excised IESs have a strong tendency to overlap each other. Among 2,172 IESs, 583 overlap at least one other IES. The overlapped IESs always share a boundary sequence (within IES), and generally have a length difference divisible by three (85.08%). Contig 8469 (a single-gene chromosome) is shown as an example (Fig. 4A). Here, 5 IESs share the same 5′ and 3′ boundary motif (5′: GCTCAAC, 3′: TCAACAG), although they are in different locations. The pointer-sequences of these IESs contain the 3′ boundary motif TCAACAG and can be represented by the motif “N(3–5)GGNTCAACAG” (where N represents A/T/G/C) (48). All length differences between pairs are divisible by three, which means the deletion and retention of any one of them retains the same reading frame (Fig. 4A). Although this alternative IES deletion at the DNA level resembles alternative intron splicing at the RNA level, and may increase the diversity of protein sequences, the functional and evolutionary significance of this is unclear, and the possibility that such variants simply reflect cellular accidents cannot be ruled out.

**Widespread, low-frequency spliced introns.** By mapping all RNA-seq reads to the genome assembly, we inferred 23,188 GT…AG type introns, 0.34 introns per gene on average. Compared with other eukaryotes, *Halteria* has relatively short introns, 59% of which are between 30 and 40 bp (Fig. 4B), with an average length of 57 bp. The average length of *Halteria* introns is nonetheless longer than that of Paramecium tetraurelia (25 bp) and Stentor coeruleus (15 bp) (55, 56), which are the shortest reported in any eukaryote (57). The first three nucleotides after GT have an obvious bias toward “AAG,” and the last three nucleotides before AG have a bias to “T(A|T)T” (Fig. 4C).

*Halteria* has a slight deficit of introns with lengths that are multiples of 3 (here called 3n introns). These 3n introns represent 29.3% of the total, in contrast with 37.9% and 32.8% for 3n + 1 and 3n + 2 introns, respectively. This deficiency of 3n introns in *Halteria* is less extreme than that in Paramecium tetraurelia; only 18.7% of *Paramecium* introns are reported as 3n introns, while 42.3% and 39.0% are 3n + 1 and 3n + 2 introns, respectively (55). *Paramecium* introns with 3n length tend to cause premature translation termination (PTC) in the event of intron retention (59.2%) (55), whereas in *Halteria*, only 12.8% of 3n introns contain at least one in-frame stop codon. *Halteria*, and possibly other species with compact genomes, may take advantage of these non-PTC-inducing 3n introns to increase the diversity of coding information.

Composite analysis in 9,060 single-gene coding nanochromosomes of *Halteria* revealed the positions of introns to be preferentially skewed toward 5′ ends (Fig. 4D). Applying the same analysis to the published genome (GCA_000295675.1) and RNA-seq (SRR578166) data sets of *Oxytricha trifallax* reveals a similar distribution of intron positions in the two species (Fig. 4D), indicating that 5′ skew of introns may be common in ciliates with nanochromosomes. This distribution may result from different selective pressures on introns at different positions in nanochromosomes. For example, assuming that ciliates have a distance-based mechanism of nonsense-mediated decay (NMD) to selectively degrade prematurely terminated mRNAs, the establishment of the first intron may modify the selective environment of downstream intron colonization events if the first intron already covers a large fraction of the total potential for NMD (58).

In contrast with the limited intron alternative splicing found in Paramecium tetraurelia (where 0.9% of 13,498 introns are alternatively spliced) (55, 59), *Halteria* exhibit a larger incidence of alternative splicing during transcription, where 3,593 introns (15.5%) overlap at least one other intron in 1,475 genome locations. Of these, 3,130 of 3,593 alternatively spliced introns share a donor (GT) or acceptor (AG) site with at least one other intron, and there is a strong preference for sharing the same donor (GT) rather than acceptor (AG) site.

By analyzing the length difference between pairs of introns sharing the same donor or acceptor site, we found that 66.84% of the additional sequence between two overlapped introns is divisible by three, which is much higher than the random expectation of 33.33%, and the proportion of 3n introns among total introns, which is 29.3%. This implies that the removal of alternatively spliced introns in this species tends to leave the original reading frame intact.

Finally, we quantified the mapped RNA-seq reads for all mRNA isoforms to determine the major isoform (the most abundant spliced variant) in each intron location. On average, the frequency of the minor form is 7.7% of the major form. Again, although this large-scale alternative mRNA splicing, along with alternative IES deletion, may increase the proteomic complexity, it remains unclear whether this is adaptive, maladaptive, or effectively neutral (43, 44).

### Transcriptional initiation and termination in tiny chromosomes.

**FIG 5:**
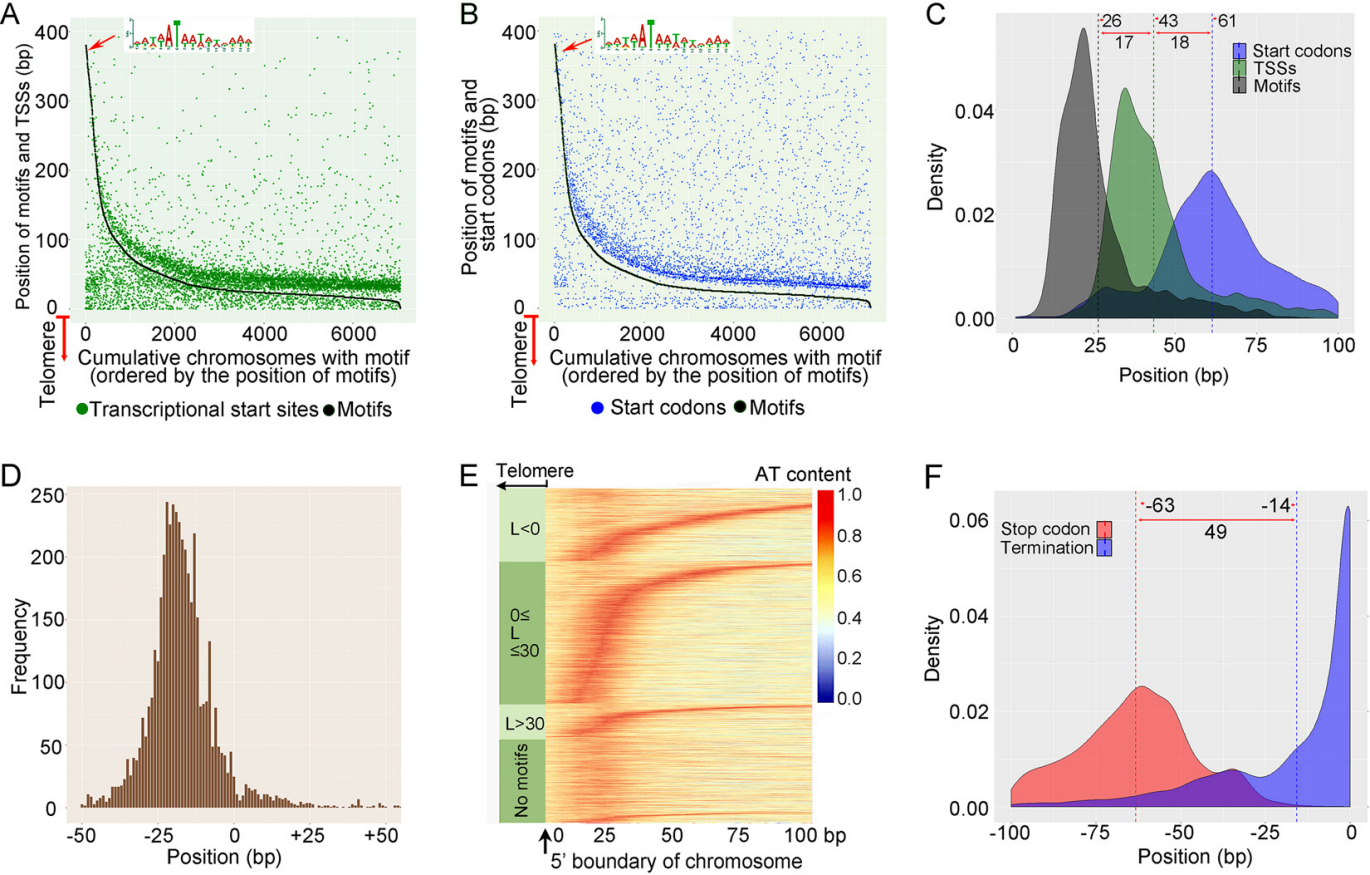
Transcription initiation and termination in tiny chromosomes. (A) Locations of TATA box-like motif and transcription start site in each of 7,061 single-gene chromosomes measured from the end of the 5′ telomere. On the *x* axis, from 0 to 7,061, all 7,061 single-gene chromosomes are ordered by decreasing distance of the TATA box like motifs from the telomere. The *y* axis denotes the position of motifs (black dots, joined as a line) or transcription start sites (green dots); 0 on the *y* axis represents the 5′ boundary of the chromosome (i.e., the motif is adjacent to the telomere). (B) Locations of TATA box-like motifs and translation start codons in each single-gene chromosome measured from the end of the 5′ telomere, as in Fig. 5A (C). The frequency distributions of positions of translation start codons (blue), transcription start sites (green), and TATA box-like motifs (gray), measured as the distance from the 5′ boundary of chromosomes (telomeres excluded). The dashed lines indicate the mean values. (D) Frequency profile of TATA box-like motifs within the region from −50 to +50 relative to the TSS (0). (E) Sliding-window analysis of AT content of 100-bp 5′ subtelomeric regions of single-gene coding chromosomes separated into four groups (based on the position of the TATA box-like motif and first mapped RNA read). The window size for calculating the AT content is 20 bp. The 0-bp location on the *x* axis is the 5′ boundary of the chromosome after removing telomeres. Each row represents a chromosome. In each group, chromosomes are ordered by the position of their motifs; the group without a TATA box-like motif is in random order. (F) The frequency distribution of translation stop codons (red) and transcription termination sites (blue), measured as the distance from the 3′ boundary of the chromosome (telomeres excluded). The dashed lines indicate the mean values.

**Transcriptional initiation.** After removing the telomeres, the 5′ subtelomeric regions of the coding strand of single-gene coding chromosomes, which are potential locations for transcription-start sites, were searched for potential regulatory sequence motifs using the MEME software (60). A conservative 15-bp motif region was discovered in 7,061 of 9,060 (78%) single-gene chromosomes (Fig. 5A and B). This AT-rich motif has an invariant T residue at position +6 and resembles the “TATA box” structure known in other organisms. Of these motifs, 75% are located within 30 bp of the 5′ telomere, the average distance being just 26 bp (Fig. 5C). To determine whether this motif is related to transcription initiation, we mapped RNA-seq reads to all single-gene-coding chromosomes, obtained the mapping boundary of RNA-seq reads to estimate the transcription-start site, and plotted them with the positions of motifs (Fig. 5A). We also plotted the position of the predicted translation start codon in each single-gene chromosome with the position of the TATA box-like motifs (Fig. 5B). As shown in these two figures, the position of this motif is highly correlated with the locations of both the transcription start site and the translation initiation codon. Meanwhile, this motif is not detected in the 3′ region. These results are consistent with our supposition that the motif is more likely to be transcription-initiation related. These TATA box-like motifs may function like transcription-factor-binding sites to initiate transcription.

Analysis of the distances between motifs and transcription start sites (TSSs) revealed 88% of them to be <30 bp in length. According to the distance L = P_TSS_ − P_motif_, where P_TSS_ is the position of the TSS, and P_motif_ is the position of the start of the motif, the 7,061 chromosomes with a motif are approximately divided into three groups: L < 0 (TSS located upstream of the motif region), 0 ≤ L ≤ 30 (TSS located <30 bp downstream of the motif region), and L > 30 (TSS located >30 bp downstream of the motif) (Table 3).

**TABLE 3:** Numbers and proportions of orthologs between *Haltera grandinella* and two other unicellular eukaryotic organisms (*Oxytricha trifallax* and Tetrahymena thermophila)

Group^*a*^	No.	Annotated genes	*Oxytricha trifallax*	Tetrahymena thermophila	TPM^*b*^
L > 30	911	0.68	0.53	0.29	5.12
0 ≤ L ≤ 30	3,876	0.78	0.71	0.38	26.74
L < 0	1,959	0.80	0.74	0.43	134.44
No motif	2,002	0.12	0.21	0.05	29.57

a RNA-seq coverage of four groups of chromosomes. L is the distance between the position of motifs (P_motif_) and the inferred position of transcriptional start sites (P_TSS_), defined by L = P_TSS_ − P_motif_. No motif indicates the absence of an obvious TATA box-like motif in the nanochromosome.

b TPM, transcripts per million.

The L > 30 group has the lowest RNA-seq coverage in *Halteria*, which is consistent with the high P_TSS_ value for this group being potentially biased upward due to low expression, resulting in deficient read mapping, i.e., the P_TSS_ value in the L > 30 group may not reflect real transcription start sites. Notably, a relatively small fraction of the genes in the L > 30 group have orthologs in other ciliates (*Oxytricha trifallax* and Tetrahymena thermophila) (compared with the L < 0 and 0 ≤ L ≤ 30 groups), suggesting that the nonconserved genes tend to have lower expression levels (Table 3). In contrast, the group L < 0 had the highest RNA-seq coverage and proportion of phylogenetically conserved genes. The group 0 ≤ L ≤ 30 and the group without motifs have similar RNA-seq coverage, near the average level of transcripts per million (TPM) for all chromosomes, suggesting that even though the conserved TATA box-like motif observed in most chromosomes is related to transcription initiation, it is not essential for transcription. The group without motifs had the lowest proportion of orthologs and functionally annotated genes, suggesting that chromosomes without this TATA box-like motif may harbor genes specific to *Halteria*.

The group 0 ≤ L ≤ 30 (3,876 chromosomes) was selected for further analysis of the locations of the TSSs, TATA box-like motifs, and translation start codons. As shown in Fig. 5C, the TATA box-like motifs start on average at 26 bp after the 5′ telomere, with the TSSs starting on average at 17 bp downstream of the motifs and translation start codons on average at 18 bp downstream of TSSs, indicating an average 5′ UTR of only ∼18 bp in length. Compared with TATA boxes of other reported eukaryotes, those in the *Halteria* genome distribute in a narrow area and much closer to TSSs (−25 to −13 bp, Fig. 5D). In the model unicellular yeast Saccharomyces cerevisiae, TATA boxes mainly distribute between −120 and −40 bp relative to TSSs (61). In humans, where the sizes of intergenic regions are expanded greatly, the distribution of TATA boxes is even wider, from −1,000 to +1,000 bp relative to TSSs (61). The extremely short chromosomes of *Halteria* greatly limit the distribution of TATA boxes, but still the regular relative spatial distribution is striking.

To further investigate transcript structures and detect potential TATA box regions, we conducted a sliding-window analysis of the A/T composition in all 100-bp 5′ subtelomeric regions (Fig. 5E). Compared with chromosomes with a clear TATA box region, the chromosomes without motifs still possess AT-rich regions with lengths of <25 bp and distributed in similar positions as typical motif regions, suggesting that these *Halteria*-specific genes may be in the evolutionary process of forming or losing a stable TATA box. Based on previous research of anomalous base compositions in subtelomeric regions of *Oxytricha* (62), the high AT composition may also act as a target for proteins responsible for excision of micronuclear sequence. By creating a differential chromatin structure along micronuclear DNA, the high AT composition might affect DNA structure to produce a specific excision target during the formation of the macronuclear genome.

**Transcription termination.** The RNA-seq reads mapped to all single-gene-coding chromosomes were also used to obtain the position of the last mapped RNA-seq read, revealing a simple pattern for the termination of transcription (Fig. 5F); 34% of chromosomes terminate transcription at the last nucleotide before the telomere, and 75% within the last 25 bp before telomeres. The mean value of the position of a transcription termination site is 14 bp before telomeres. This suggests that transcription termination in single-gene chromosomes of *Halteria* may simply occur when the RNA polymerase reaches the ends of nanochromosomes. Most stop codons located at 63 bp before telomeres, indicating a mean 3′ UTR length of ∼49 bp (Fig. 5F). Unlike the 5′ UTR, no conserved motif was identified in the 3′ UTR region. On average, the 3′ UTR is longer than the 5′ UTR and has a higher length variation (standard deviation of 25 bp versus 19 bp) (Fig. S5), which may be attributable to the simple pattern of transcriptional termination.

In conclusion, the polyploid macronuclear genome of *Halteria* closely parallels the transcriptome, comprising ∼23,000 haploid gene-sized nanochromosomes, the longest and the shortest being 74,042 bp and 345 bp, respectively. The macronuclear copy numbers of nanochromosomes are nonuniform and correlate with gene expression levels, implying a potential connection between the chromosomal copy number and the level of transcription, although the functional significance of this correlation remains unclear. Further analyses combining genomic and transcriptomic data sets indicated that large-scale alternative deletions occur during macronuclear formation (IES deletion) and transcription (intron deletion). These alternative deletions may produce functional gene products, but may also simply reflect erroneous splicing. Finally, the analyses of transcriptional initiation/termination of nanochromosomes reveal conserved TATA box-like motifs and short untranslated regions (∼10 bp) in the 5′ subtelomeric regions, and extremely compact untranslated 3′ regions (∼49 bp). The mean UTR lengths of *Halteria* resemble those reported in *Oxytricha trifallax* (20), making the gene structures in *Halteria* and *Oxytricha* the most compact known among eukaryotes.

10.1128/mBio.01964-20.5 FIG S5 Length distribution of the 5′ UTR and 3′ UTR in nanochromosomes. Download FIG S5, TIF file, 0.4 MB. Copyright © 2021 Zheng et al. 2021 Zheng et al. This content is distributed under the terms of the Creative Commons Attribution 4.0 International license.

## MATERIALS AND METHODS

### Cell isolation and culture.

A *Halteria grandinella* cell was isolated from a freshwater pond in Baihuayuan Park (36°04’N, 120°22’E), Qingdao, China, and was identified by its morphological features and small subunit ribosomal ribonucleic acid (SSU-rRNA) gene sequence. A single cell was picked, washed, and cultivated in cell culture flasks with filtered and autoclaved pond water. All downstream experiments, including DNA and RNA extractions, were based on clones produced by asexual reproduction of this clone-founding cell. Escherichia coli strain DH5α was applied as the food source. The ciliate culture for DNA and RNA extraction was cultivated for 7 days at 23°C.

### DNA extraction, RNA extraction, and Illumina sequencing.

Before the DNA and RNA extraction, cells were starved for 2 days and ampicillin was added to reduce contamination by E. coli and other bacteria. Cells were harvested by centrifugation at 300 × *g* for 5 min. DNA was extracted using phenol chloroform extraction followed by ethanol precipitation. RNA extraction was performed with the RNeasy Plus minikit (Qiagen, Germany) following the manufacturer’s instructions.

The DNA library was constructed with NEBNext DNA library prep master mix set for Illumina (New England BioLabs [NEB], USA) following the manufacturer’s instructions. High-throughput sequencing was performed on an Illumina Hiseq 2500 platform. The paired-end sequencing produced 20G of clean data after quality control and removing adapters (read length: 150 bp).

The RNA libraries were generated using NEBNext Ultra RNA library prep kit for Illumina (NEB, USA) following the manufacturer’s instructions. Four RNA-seq replicates were sequenced. High-throughput sequencing was performed on Illumina Hiseq 2500 platform and produced 10G of clean data for each RNA library after quality control and removing adapters (read length: 150 bp).

### Genome assembly.

Raw sequencing reads were trimmed using Trimmomatic with the following parameter: “ILLUMINACLIP:adapter_path:2:30:10 LEADING:10 TRAILING:10 SLIDINGWINDOW:4:20” (63). The genome assembly was performed with a combination of the SPAdes and MaSuRCA assemblers (64, 65). The MaSuRCA assembler was run with the default parameters. The super-reads output file from the MaSuRCA assembler was applied to the SPAdes assembler as reference. The SPAdes assembler was run with the following parameters: -k 21,33,55,77 –careful –trusted-contigs <super-reads file>. SPAdes software generated 52,353 contigs. The output contigs from SPAdes were split into three files (0/1/2-telomere) according to the number of telomeres. Contigs containing *Halteria* telomere are considered high-quality and were retained without contamination filtering. Contigs without telomeres were filtered based on the GC content. Contigs with GC content higher than 60% were considered contamination and were removed. Contigs with no telomeres, low sequencing coverage (coverage < 2×), and lengths < 500 bp were considered low quality, and were removed.

Fifty thousand randomly selected bacterial genomes downloaded from the NCBI database were used as the target database. The filtered genome assembly was split into 426,052 150-bp-long fragments and aligned with the target database. No fragment was mapped to the bacterial database, indicating that this genome assembly does not contain chimeric contigs resulting from false recombination between *Halteria* and bacterial genome.

The micronuclear (MIC) sequence contamination was estimated by checking the sequencing coverage of all sites using RSEM software (66). Genomic regions with less than 5% sequencing coverage of its adjacent regions were considered MIC contamination and removed. No such region was detected in the final assembly.

QUAST was used to obtain the GC content, *N*_50_, and other genomic statistics (67). RSEM software, which is based on bowtie2, was used in the calculation of sequencing depth of each contig (66).

### Gene prediction and annotation.

The RNA-seq reads and genome contigs were aligned using Tophat2 to predict introns (68). Before gene prediction, all introns were removed from the genomic contigs. After removing introns, intron-free contigs were aligned with the Swiss-Prot database using blastx (E value = 1e−5, querygenecode = 6) to identify genes in our assembly (30). A perl script “blast_xml_protein_coding_sequence_extract_v2.pl” (supplemental material appendix, Code S3) was used to analyze the “.xml” output file of blastx (using contigs of the *Halteria* genome as the query and Swiss-Prot as the target database). In this script, all matched genes in the Swiss-Prot database for each query (contigs of *Halteria* genome) were identified and extracted by searching for the start and stop codons of the longest potential open reading frame. The output file of this script was used in the model training of AUGUSTUS software. Detailed functional information of each gene was obtained using a perl script “entrez_information_extract.pl” (supplemental material appendix, Code S4).

The *de novo* gene prediction was performed using AUGUSTUS (version 2.5.5) (69). The AUGUSTUS gene model of *Halteria* was trained using the original genome assembly and genes models predicted by Code S3. In the model training of AUGUSTUS software, 5,000 genes generated by Code S3 were randomly picked as the training data set. Training of the gene prediction model was repeated 19 times until the sensitivity and specificity stabilized at the highest level (89% and 66% at nucleotide level, 62% and 51% at exon level, and 42% and 41% at gene level, respectively). In the file containing parameters, the conventional TAA and TAG codons were modified to encode glutamine instead of the translation stop codon, as in other hypotrichous ciliates (*Oxytricha* and *Stylonchyia*) (70). Because the default minimum intron length of AUGUSTUS is too long for ciliates, the parameter <min_intron_len> in the source code file <type.cc> in AUGUSTUS was modified from 39 to 15 to recompile the software. RNA-seq reads were assembled into transcripts using rnaSPAdes (71). These transcripts were then aligned with the genome assembly by BLAT to produce the “hints” file for AUGUSTUS gene prediction (72). The predicting result of AUGUSTUS was filtered to discard the genes without start and stop codons using a perl script “gff_analysis.pl” (supplemental material appendix, Code S5). The remaining protein-coding genes predicted by AUGUSTUS were aligned with the Swiss-Prot database to add functional information (73).

The *de novo* predicted genes based on AUGUSTUS and predicted genes based on the Swiss-Prot database were combined to form the final result of gene predictions. Another perl script “combine_database_and_denovo_annotation.pl” (supplemental material appendix, Code S6) was designed to unite the genes predicted by the Swiss-Prot database and by AUGUSTUS. For the genes shared by both predictions, those predicted by the Swiss-Prot database were retained in the final prediction file. The alignment between all predicted genes and the COG (Clusters of Orthologous Genes) database was performed using blastx method with default parameters (74).

### Polymorphic sites detection.

The fastq file of genomic sequencing was aligned with the genome assembly using bowtie2 (31). Samtools was used for converting sam to bam files and for generating the Mpileup file (75). Mapgd was used for converting the Mpileup file into quartet sets of reads which comprised numbers of observed As, Cs, Gs, and Ts at each site (76). The analyses of minor allele frequency and other parameters were performed using a perl script “pool_population_analysis.pl” (supplemental material appendix, Code S7). Zero-fold redundant sites are those nucleotides at which all changes are nonsynonymous.

### Alternatively spliced IES detection.

The software ADFinder was designed to filter the paired-end reads, align reads with genome assembly, and analyze the resulting alignment (unpublished software). This software is written in python and utilizes the bowtie2 software (–local, -k 5, –ma 3) in read alignments. The local mode of bowtie2 allows each read to align with the reference genome with a soft clip (alignments might be “trimmed” to optimize the alignment score). In the output file of bowtie2 software, the CIGAR field of soft-clipped reads is shown in the format “110M40S” (“110 matches + 40 mismatches”) or “110S40M” (“110 mismatches + 40 matches”), suggesting that this read is partially mapped. This kind of partially mapped read is essential in identifying the splicing event in the reference genome. Then, soft-clipped reads in alignments were used to generate the splice sites. If one read was mapped as a soft-clipped read at two or more different genomic locations, all matching records of this single read were analyzed. For example, if a read was matched to a contig at the x^th^ nucleotide with the CIGAR field “110M40S.” and this read was also mapped to the same contig for the second time at the y^th^ nucleotide with the CIGAR field “110S40M,” then the DNA sequence between x + 110 and y bp was regarded as a deletion event. Some fully mapped internal-broken reads which are end-to-end matched but contain a small deletion fragment may also be used to identify small deletion events. The CIGAR field of internal-broken read is shown in the format “50M20D80M” (“50 matches + 20 deletions + 80 matches”). For these internal-broken reads, each can simply signify a splicing event. The core code of ADFinder was attached in our source code list (supplemental material appendix, Code S2). The detailed description, purpose, and usage of ADFinder is shown in our GitHub webpage (https://github.com/weibozheng/ADFinder). The RNA and DNA sequencing coverage of each splicing site was determined by mapping reads with recombined segments produced by the following methods. The “excised” version (with the IES deleted) was inferred by the conjoining within genomic sequence of the 125-bp 5′ upstream and 125-bp 3′ downstream region of the IES. The “nonexcised” version was inferred in two ways according to the length of IES: (i) if the presumed IES length x was <250 bp, then (250 − x)/2 bp 5′ upstream, x bp IES, and (250 − x)/2 bp 3′ downstream regions were combined as indicators of the “nonexcised” version (with the IES retained); and (ii) if the IES length x was >250 bp, the IES itself was treated as an indicator of the “nonexcised” version. The schematic plot of the above description is shown in Fig. S6.

10.1128/mBio.01964-20.6 FIG S6 Schematic plot of spliced/nonspliced versions of a genomic location containing an alternative IES splicing event. Download FIG S6, TIF file, 0.4 MB. Copyright © 2021 Zheng et al. 2021 Zheng et al. This content is distributed under the terms of the Creative Commons Attribution 4.0 International license.

### Transcriptional initiation analysis.

The motif searching software MEME was used for searching the motifs in 400-bp 5′ subtelomeric regions (60). Bowtie2 was used in read-mapping between all RNA-seq reads and single-gene chromosomes (31). Samtools was used for filtering and sorting the mapping results produced by Bowtie2 (75). The sorted *.sam files produced by Samtools were applied to a perl script “get_trans_ini_termi.pl” (supplemental material appendix, Code S8) to extract the positions of potential transcriptional initiation and termination sites in each chromosome. The search for homologous genes between *Halteria grandinella* and two other ciliates (*Oxytricha trifallax* and Tetrahymena thermophila) was conducted by blastx using protein identity > 30% and overlap region > 60% as criteria.

### Data and source code availability.

The final genome assembly, genomic reads, and RNA-seq reads have been deposited in GenBank (genome assembly: RRYP01000000, genomic reads: SRX5087334, RNA-seq reads: SRX5097405, SRX5097406, SRX5097407, and SRX5097408). All source codes in our research are available in this GitHub webpage: https://github.com/weibozheng/Halteria_genome_research.
